# The Alteration of Intraocular Pressure and Ocular Pulse Amplitude by Retrobulbar Anaesthesia—A Search for Risk Factors for Serious Complications Due to Retrobulbar Anaesthesia

**DOI:** 10.3390/jcm13175172

**Published:** 2024-08-31

**Authors:** Deborah Dobberstein, Berthold Seitz, Anja Viestenz, Arne Viestenz

**Affiliations:** 1 Department of Ophthalmology, Saarland University Medical Center, 66421 Homburg, Germany; berthold.seitz@uks.eu (B.S.);; 2 Department of Ophthalmology, University Medicine Halle, Martin-Luther-University Halle-Wittenberg, 06120 Halle, Germany

**Keywords:** retrobulbar anaesthesia risks, intraocular pressure change in retrobulbar anaesthesia, ocular pulse amplitude change in retrobulbar anaesthesia, eye surgery, diabetes mellitus and local anaesthesia

## Abstract

Our goal was to assess the impact of retrobulbar anaesthesia on ocular pressure and perfusion development and to find out if there were systemic or biometric parameters of patients affecting them in order to understand the effect of retrobulbar anaesthesia better. **Methods**: Changes in intraocular pressure (IOP) and ocular pulse amplitude (OPA) using a dynamic contour tonometer (DCT) were noted before and after retrobulbar anaesthesia (RBA) in combination with five minutes of oculopression at 40 mmHg in 134 patients. Only results with a quality Q 1–3 were considered for further statistical analysis. Systemic and ophthalmic parameters were noted and their impact was tested using linear regression. **Results**: IOP decreased from 18.9 ± 7.2 mmHg to 15.4 ± 6.3 mmHg (*n* = 71, *p* = 0.001) after first RBA. The dosage of midazolam administered during premedication was found to increase IOP significantly after first RBA (*B* = 3.75; *R*^2^ = 0.38). Ocular pulse amplitude decreased significantly from 3.8 ± 1.7 mmHg to 3.0 ± 1.9 mmHg after first RBA (*n* = 72, *p* < 0.001). This change was found to be dependent on the presence of diabetes mellitus (*n* = 68, *p* = 0.048). **Conclusions**: IOP and OPA decrease after RBA and oculopression. Caution is needed with midazolam premedication due to potential IOP increase. Patients with diabetes and pre-existing retinal or optic nerve damage should consider alternative anaesthesia methods, such as eye drops or general anaesthesia, due to the observed decrease in OPA after RBA and oculopression.

## 1. Introduction

Retrobulbar anaesthesia (RBA) is an established method of locally anaesthetising the eye during ophthalmological procedures. The Department of Ophthalmology at Saarland University Medical Center alone performs around 1000 RBA per year.

A decisive advantage of this form of anaesthesia is the possibility of allowing patients with many comorbidities to undergo eye surgery, especially when general anaesthesia is contraindicated. 

Although this method is routinely performed, serious complications are described in the literature, such as iatrogenic central retinal artery occlusion, combined central retinal artery, and central retinal vein occlusion [1,2]. The exact causes of these incidents are often difficult to determine. 

The main aim of this study was to investigate the effects of retrobulbar anaesthesia on intraocular pressure and blood flow in the eye in a practical setting in order to find out which biometric or constitutional parameters of the patient could have an influence on these effects. There have been no recent scientific studies specifically investigating this aspect.

This issue is timeless because the principles of patient safety and individualized medical care remain fundamental in healthcare, ensuring that anaesthetic practices continue to evolve based on ongoing research and the unique needs of diverse patient populations.

## 2. Materials and Methods

The present study is a prospective, non-randomised, observational study. Non-pregnant persons of legal age who were in full possession of their mental and physical faculties were included. The ethics vote was available for the study of intraocular pressure measurement during eye surgery (vote of the Saarland Medical Association: 144/13). Informed consent was obtained from all subjects involved in the study. Intraocular pressure (IOP) and ocular pulse amplitude (OPA) were measured using dynamic contour tonometry (DCT) “MT Swiss Microtechnology AG a Ziemer Group Company, Port, CH” before RBA and after five minutes of oculopression at 40 mmHg after RBA. 

**Figure 1:**
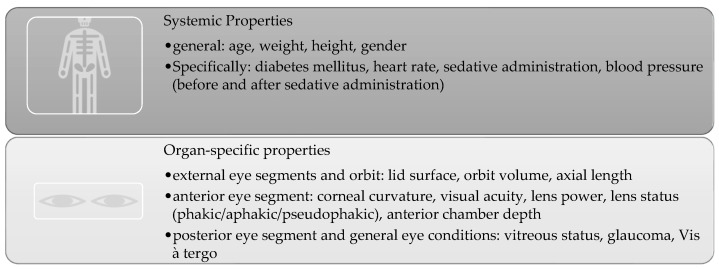
Overview of influencing factors on retrobulbar anaesthesia.

Systemic (age, weight, height, diabetes mellitus, heart rate, mean arterial pressure (MAP) before sedative administration, sedative administration and quantity) and organ-related characteristics (eyelid area, orbital volume, length of the eye (AL), IOP/OPA before RBA, lens status (phak/aphak/pseudophak), anterior chamber depth (VKT), vitreous body status, glaucoma) were determined (see Figure 1). Eyelid width, eyelid area, orbital height and orbital width were determined using a hand-held ruler based on the externally recognisable anatomical structures [3]. Orbital depth was defined by measuring the distance from the outer lid angle to the tragus base. The calculation of certain parameters was based on the following formula: Eyelid area (ellipsoidal eyelid area= π × 0.5 × eyelid width × eyelid width [cm^2^]), eye volume (eye volume [cm^3^] = 4/3 × π × (axial length [mm]/2)^3^/1000), orbital volume (orbital cone volume [cm^3^] = ellipsoidal lid area [cm^2^] × ½ orbital depth [cm]), and orbit/eye (orbit/eye = orbital volume [cm^3^]/eye volume [cm^3^]). 

Keratometer values, anterior chamber depth (VKT), and axial length (AL) were determined by optical biometry [4]. Visual acuity sc/cc and refraction were determined preoperatively. A history of previous eye surgery and ophthalmologically relevant diseases such as glaucoma or diabetes mellitus was taken from each patients’ history. Blood pressure and heart rate were measured preoperatively as part of continuous monitoring. 

All measurements for the determination of IOP, OPA, and measurement quality were performed with the DCT in the supine patient [5,6,7,8,9,10,11]. The vis-à-tergo (VAT) was determined by the surgeon during the procedure. It indicates how much pressure is present in the vitreous body. The higher the vis-à-tergo, the higher the pressure in the vitreous body and the higher the risk of complications. A scale from 0 to 3 was defined for VAT, as explained in Table 1 [12].

The measurement took place in the preparation room of the operating theatre and after general measures (checking the patient’s identity, surgical indication, creation of an antebrachial access, administration of premedication if required), oxybuprocaine eye drops were administered to the eye to be measured and later operated on. The RBA was performed by an ophthalmological surgeon experienced and trained in this form of anaesthesia (AV) [1]. The RBA solution was composed as follows: 150 I.U. hyaluronidase dissolved in 3.5 mL bupivacaine hydrochloride 0.75% and 1.5 mL articaine 2% (UKS-SOP). In order to fulfil the required conditions for measurement with the DCT, the sensor head of the DCT must be aligned parallel to the floor and perpendicular to the globe. For this purpose, the patient’s head was turned almost 90° towards the examiner or towards the measuring eye and the DCT was handheld [11]. Measurements were taken before and after RBA and 5 min of oculopression at 40 mmHg [13]. If the patient was scheduled for retinal surgery or if the first RBA did not achieve the required akinesia, a second RBA was given (*n* = 35). This resulted in up to three measurements: before the first, after the first, and after the second RBA (if a second RBA was required).

The anonymised data were analysed using SPSS for Windows 20.1 (IBM Corp. Released 2021. IBM SPSS Statistics for Windows, Version 28.0. Armonk, NY, USA: IBM Corp).

Firstly, the change in IOP/OPA was tested for statistical significance. The further analysis focused exclusively on those undergoing only one RBA due to the small size of the patient group with two RBAs. All influencing variables that were found to be statistically significant in the univariate linear regression were then further analysed using multiple linear regression with forward selection. As no linear relationship can be assumed for VAT, bivariate correlation was used to test for a statistically significant dependency. 

## 3. Results

### 3.1. Descriptive Statistics

#### 3.1.1. Patient Cohort

A total of 134 patients’ eyes were measured. Non-pregnant persons of legal age who were in full possession of their mental and physical faculties and who presented for ophthalmic surgery with retrobulbar anaesthesia at the Department of Ophthalmology at Saarland University Medical Center were included in a continuous, non-randomised manner. 

A total of 56 right and 78 left eyes were measured. A total of 57 patients were men and 77 women. The mean age of the subjects was 72.1 ± 11.4 years. A total of 91 patients came for cataract surgery [KAT] and 12 for pars plana vitrectomy [PPV]. The remaining 31 patients underwent combined surgery [KAT + PPV]. 

The mean height was 168 ± 10 cm. The median body weight was 78 ± 16 kg. This resulted in an average BMI in the pre-adipose range of 28 ± 5 kg/m^2^. The mean arterial blood pressure (MAP) before midazolam administration was 91 ± 14 mmHg. The mean heart rate was 74 ± 15 beats/minute. The medical history revealed that 24% had diabetes mellitus and 12% had glaucoma.

The median visual acuity of the eyes examined was 0.4 according to Snellen. The majority of eyes were phakic (*n* = 110), 24 eyes were pre-operated, with 23 patients being pseudophakic. The anterior chamber depth showed a mean depth of 3.2 mm. The average axial length of all eyes was 23.4 mm (Table 2).

#### 3.1.2. Validity of the Measurements with the DCT

The validity Q of the measurements with the DCT was defined by the manufacturer on a scale of 1–5. All values ≤ 3 have a good probability of validity, all values > 3 could have been falsified by confounding factors [9,10,11]. The examination of the measured values in this study for IOP and OPA showed a significant group difference between values of validity Q 1–3 and Q 4–5 (Table 3). The measured values of a validity Q 4–5 are potentially unreliable. Therefore, in the following we always refer to measured values with a validity Q ≤ 3. 

#### 3.1.3. Change in IOP

**Figure 2:**
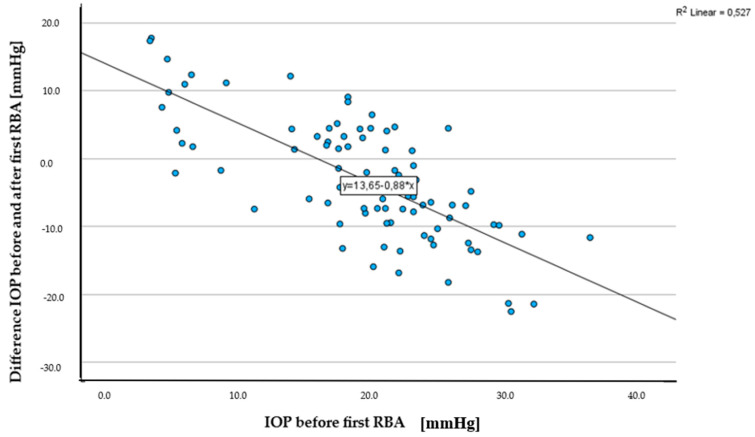
Scatterplot IOP before first RBA [mmHg] and difference in IOP before and after first RBA [mmHg].

The IOP before the first retrobulbar injection averaged 18.9 ± 7.2 mmHg with a minimum of 2.9 mmHg and a maximum of 35.8 mmHg. After the first injection, there was a decrease to an average of 15.4 ± 6.3 mmHg, which then rose again to 17.5 ± 8.5 mmHg after the second injection (Table 4). The change in pressure after the first RBA (Figure 2) was statistically significant (Wilcoxon test, *p* = 0.001), while the change in IOP between the first and second RBA (paired *t*-test for connected samples, *p* = 0.32) or before the first and after the second RBA (Wilcoxon test, *p* = 0.41) was not significant.

#### 3.1.4. Change in OPA

**Figure 3:**
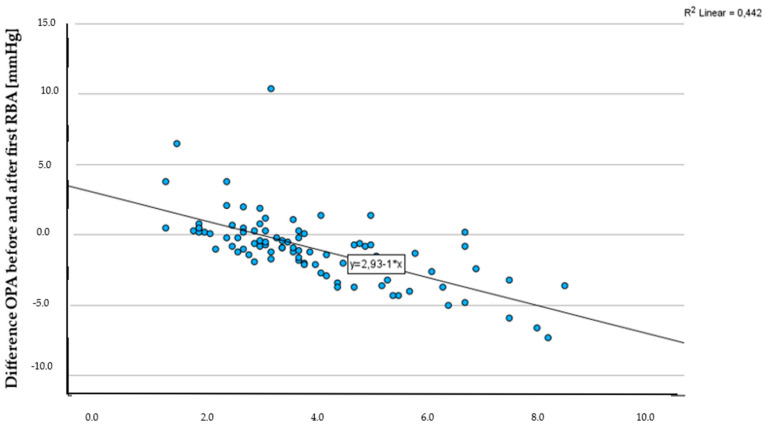
Scatterplot OPA before first RBA [mmHg] and difference in OPA before and after first RBA [mmHg].

The OPA before the first retrobulbar injection was measured with an average of 3.8 ± 1.7 mmHg, with a minimum value of 1.2 mmHg and a maximum value of 8.4 mmHg. Following the administration of the first injection, OPA decreased to an average of 3.0 ± 1.9 mmHg, and further declined to 2.6 ± 1.6 mmHg after the second injection (Table 5). The observed development in OPA before and after the first retrobulbar anaesthesia (Figure 3) was found to be statistically significant (paired *t*-test for related samples, *p* < 0.001). However, there were no significant changes in OPA between the first and second RBA (paired *t*-test for related samples, *p* = 0.93), or before and after the second RBA (paired *t*-test for related samples, *p* = 0.28).

### 3.2. Factors Influencing IOP/OPA

Lens status, vitreous body status, glaucoma, heart rate, age, and VAT 2-3 had no influence on the change in IOP and OPA. All other parameters were further analysed using multiple linear regression with forward selection (Table 6).

#### 3.2.1. IOP

IOP experienced the most significant decrease after a single retrobulbar anaesthesia (*B* = 0.5; *R*^2^ = 0.3). Additionally, the IOP exhibited the largest increase in response to the dosage of midazolam administered (*B* = −3.8; *R*^2^ = 0.4). During a second RBA, it was observed that the IOP decreased as the axis length increased (*B* = 2.9; *R*^2^ = 0.6). This suggests that there is a relationship between axis length and changes in IOP during subsequent RBAs (Table 7). Interestingly, the change in IOP between the first and second RBA was found to be influenced by the initial IOP measurement before the first RBA (*B* = 1.0; *R*^2^ = 0.6).

#### 3.2.2. OPA

The OPA decreased after a single RBA depending on the OPA before the first RBA (*B* = 0.6; *R*^2^ = 0.3) and diabetes mellitus (*B* = 0.7; *R*^2^ = 0.4). After a second RBA, the MAP before midazolam administration (*B* = 0.1; *R*^2^ = 0.6) and the OPA before the first RBA (*B* = 0.8; *R*^2^ = 0.5) had the greatest influence on the development of the OPA (Table 8). The change in OPA after the first and second RBA was most strongly influenced by the MAP before midazolam administration (*B* = 0.1; *R*^2^ = 0.5).

## 4. Discussion

Excessive IOP changes and decreased OPA could be potential risks in retrobulbar anaesthesia. Both significantly decrease after a single procedure. Following two procedures, OPA continues to decline. Systemic factors are influential. Notably, OPA decreases substantially in patients with diabetes mellitus. Detailed discussion of the study’s findings on IOP and OPA follows, considering current scientific knowledge.

### 4.1. IOP

Previous studies [13,14,15,16,17] have described an increase in intraocular pressure due to RBA. However, these values always refer to a pressure measurement before oculopression. If the IOP is measured after oculopression, oculopression is said to have a pressure-lowering effect [13,18,19]. Therefore, the decrease in IOP in this study is most likely explained by the oculopression that took place. To gain a better understanding of these relationships, it would be useful to measure IOP immediately after RBA. This should be taken into account in future studies analysing potential risk factors.

The change in IOP after a single RBA depends on the IOP before RBA and the amount of midazolam administered. The former expresses the pressure-lowering effect of the RBA per se; the latter is remarkable, as the quantities of midazolam (max. 1.3 mg) hardly suggest a systemic effect. To the best of our knowledge, no studies exist that have investigated the effects on IOP in a comparable patient population at this dosage. At least some studies indicate that midazolam may not lower IOP [20,21]. It is possible that the central muscle relaxant effect of midazolam in this dosage range impairs the outflow of ventricular fluid through the Schlemm canal. However, it is important to note that our study does not provide sufficient evidence to draw conclusions about the underlying mechanisms and the significance level of this influence is relatively low. Further research is needed to explore this topic in more depth. It would also be interesting to investigate alternative agents for premedication regarding their effect on IOP and OPA undergoing RBA, such as dexmedetomidine.

It turned out that with the second injection, the eye pressure dropped significantly in eyes with a longer axial length. We explained this phenomenon by ocular rigidity (OR). Detorakis [22] described the OR as an interplay of pressure and volume ratios in a space bound by elastic walls with non-compressible contents. In many studies, an inversely proportional relationship between axial length of the eye and OR was observed, i.e., large eyes had a lower OR than small eyes [23,24]. Clement et al. [25] defined the IOP as the sum of the aqueous humour production/discharge ratio and the episcleral venous pressure. Theoretically, additional extraocular pressure on the eyeball would initially increase the IOP. To compensate for this, the outflow of intraocular fluid into episcleral veins increases or the episcleral venous pressure decreases, so that the IOP decreases [25]. This would also explain the observed significant dependence of IOP on axial length. Thus, large eyes could better absorb an increase in extraocular pressure due to a lower OR. Therefore, a second RBA should be favoured in eyes with a large axial length if lower intraocular pressures are to be aimed for.

### 4.2. OPA

The findings of the present study provide valuable additional insights that complement the results of previous research, which consistently reported a sustained reduction in ocular blood flow following RBA or peribulbar anaesthesia (PBA) [15,26,27,28] as a consistent decrease in OPA with each RBA procedure was observed. The observed consistent decrease in OPA with each RBA procedure aligns with the close relationship between ocular blood flow and OPA through ocular rigidity [29]. Furthermore, the significance of these findings becomes evident when considering that the decrease in OPA was not only observed after multiple RBA procedures but became significant after a single RBA. This highlights the immediate impact of RBA on ocular perfusion pressure and suggests that even a single administration of retrobulbar anaesthesia can lead to a notable reduction in ocular blood flow. Especially concerning could be the reduction in ocular blood flow after RBA in patients with diabetes mellitus. This study revealed a significant and substantial decrease in OPA among diabetics following the procedure after first RBA. Langham et al. [30] observed a change in ocular perfusion in diabetic patients with progressive diabetic retinopathy.

Another study, on the other hand, found no effect on OPA in diabetics regardless of the degree of retinopathy in a larger population [31]. To our knowledge, the change in OPA with RBA and oculopression in diabetics has not yet been investigated. Although the significance level was reached in our study, it is crucial to conduct further research with a larger sample size and consider additional factors such as the type of diabetes and patients’ health history. This would provide more comprehensive insights into the relationship between OPA and diabetes mellitus.

The influence of systemic parameters on OPA changes appears to be substantial. Specifically, alterations in systemic parameters, such as mean arterial pressure prior to RBA, have been found to correlate with a decrease in OPA following a second RBA. The recommendation of a second RBA should therefore be taken with caution if there is an increase in MAP before the procedure. These findings suggest that ocular perfusion may possess a robust autoregulatory capacity. However, it is imperative to conduct further research with a larger sample size to validate these findings before drawing conclusions for clinical practice.

### 4.3. Limitations of the Study Structure

In this context, there is potential for improvement in our study. The number of cases was low, particularly when analysing the second RBA. The measurement in the preoperative setting was often challenging and subject to a certain time pressure. The orbital measurements were approximations; measurement by MRI would be desirable. The measured values for IOP and OPA were always determined after oculopression, meaning that oculopression itself could be a confounder. Furthermore, the measured values of IOP in particular could have been influenced by the use of local anaesthetic eye drops and mydriatic agents, as well as the order of application [32,33,34,35,36]. Additionally, changes in OPA may partially result from the vasoconstrictive side effects of amide-type local anaesthetic drugs used in the RBA solution [37]. Ultimately, the methodology did not differentiate oculopression, which is routinely performed after RBA, during the monitoring of IOP and OPA. While this may affect the results, it was deemed necessary for evaluating RBA in a practical, “real-life” setting rather than under controlled conditions.

### 4.4. Summary

IOP and OPA decrease after a single RBA and oculopression. The factors associated with single retrobulbar anaesthesia in particular (dosage of midazolam/diabetes) are statistically significant and could represent important risk factors for complications during retrobulbar anaesthesia, both in terms of IOP and OPA. It is therefore advisable to consider these factors when selecting the appropriate anaesthesia method in everyday clinical practice.

### 4.5. Notes by the Authors

RBA is a not uncontroversial form of anaesthesia in ophthalmic surgery, and has been associated with serious complications ever since its use began [1,2,38,39]. In our study, none of the 134 patients experienced severe local or systemic complications. The application of RBA with subsequent 5 min oculopression as described by us has proven successful in everyday clinical practice. We observed rather mild local complications such as haematoma formation, dysaesthesia, or protracted anaesthesia. The extent to which this observation corresponds with those of other ophthalmic surgery centres and whether the technique of RBA or the use of oculopression can significantly reduce complication rates should be the subject of further investigations, taking into account the development of IOP and OPA.

## 5. Conclusions

This study contributes to our understanding of the effects of RBA on ocular hemodynamics and emphasizes the importance of considering OPA as a crucial factor in assessing the impact of anaesthesia on ocular blood flow. Premedication with midazolam should be administered taking into account a possible increase in IOP. A different form of anaesthesia (e.g., drops, general anaesthesia) is recommended, particularly in patients with a vascularly pre-damaged retina/n. opticus because of diabetes mellitus, due to the observed drop in OPA.

## Figures and Tables

**Table 1:** Definition of vis-à-tergo (VAT) [12].

VAT	Explanation
0	No increased pressure from behind
1	Slightly increased pressure with pulsation of the iris-lens diaphragm
2	Increased pressure with pulsation of the iris up to the cornea
3	Significantly increased pressure with prolapse of intraocular tissue

**Table 2:** Mean ± SD and range of body height [cm], weight [kg], body mass index (BMI) [kg/m^2^], mean arterial pressure (MAP) [mmHg], heart rate [beats/min], visual acuity, spherical equivalent (SÄQ) [Dpt], mean corneal power (K) [Dpt], anterior chamber depth [mm], and axial length (AL) [mm].

Parameters	Mean ± SD	Range from–to
Body height [cm], *n* = 132	168 ± 10	145–198
Weight [kg], *n* = 132	78 ± 16	45–140
BMI [kg/m^2^] *n* = 132	28 ± 5	17–56
MAP [mmHg] before midazolam, *n* = 127	91 ± 14	61–127
Heart rate [beats/min], *n* = 131	74 ± 15	48–118
Visual acuity after Snellen, *n* = 131	0.4 ± 0.3	0.0–1.0
SÄQ [Dpt], *n* = 126	1.0 ± 4.7	−14.4–25.4
K [Dpt], *n* = 128	43.3 ± 1.3	39.3–46.7
Anterior chamber depth [mm], *n* = 123	3.2 ± 0.7	2.0–6.3
AL [mm], *n* = 129	23.4 ± 2.2	15.2–33.8

**Table 3:** Mean ± SD [minimum–maximum] of intraocular pressure (IOP) and ocular pulse amplitude (OPA) [mmHg] before the first, after the first, and after the second retrobulbar anaesthesia (RBA) with a measurement quality of Q1–5 and Q1–3.

Time Point	Measurement	Q1–5 Mean ± SD [Minimum–Maximum] *n*	Q1–3 Mean ± SD [Minimum–Maximum] *n*
Before 1st RBA	IOP [mmHg]	17.3 ± 7.7 [1.7–35.8] *n* = 133	18.9 ± 7.2 [2.9–35.8] *n* = 88
	OPA [mmHg]	4.2 ± 2.0 [1.2–10.7] *n* = 133	3.8 ± 1.7 [1.2–8.4] *n* = 88
After 1st RBA	IOP [mmHg]	15.8 ± 6.3 [0.3–29.7] *n* = 134	15.4 ± 6.3 [0.3–29.7] *n* = 101
	OPA [mmHg]	3.0 ± 2.0 [0.6–13.5] *n* = 133	3.0 ± 1.9 [0.6–13.5] *n* = 101
After 2nd RBA	IOP [mmHg]	17.6 ± 8.6 [3.3–38.8] *n* = 35	17.5 ± 8.5 [3.3–34.1] *n* = 24
	OPA [mmHg]	2.5 ± 1.7 [0.7–7.3] *n* = 35	2.6 ± 1.6 [0.8–7.3] *n* = 24

**Table 4:** Mean ± SD [minimum–maximum] of intraocular pressure (IOP) [mmHg] before first, before second, and after second retrobulbar anaesthesia (RBA).

	Before 1st RBA	Before 2nd RBA	After 2nd RBA
IOP [mmHg] Q1–3	18.9 ± 7.2 [2.9–35.8] *n* = 88	15.4 ± 6.3 [0.3–29.7] *n* = 101	17.5 ± 8.5 [3.3–34.1] *n* = 24

**Table 5:** Mean ± SD [minimum–maximum] of ocular pulse amplitude (OPA) [mmHg] before first, before second, and after second retrobulbar anaesthesia (RBA).

	Before 1st RBA	Before 2nd RBA	After 2nd RBA
OPA [mmHg] Q1–3	3.8 ± 1.7 [1.2–8.4] *n* = 88	3.0 ± 1.9 [0.6–13.5] *n* = 101	2.6 ± 1.6 [0.8–7.3] *n* = 24

**Table 6:** Overview of influencing factors on the change in intraocular pressure (IOP)/ocular pulse amplitude (OPA) due to retrobulbar anaesthesia (RBA) (“+” significant correlation demonstrated, “−“ no significant correlation demonstrated, “-------” calculation not possible).

Influencing Factors	Difference IOP [mmHg]			Difference OPA [mmHg]		
Time Point (before/after 1st/2nd RBA)						
Time Point (before/after 1st/2nd RBA)	after 1st	before 1st/after 2nd	after 2nd	after 1st	before 1st/after 2nd	after 2nd
IOP before 1st RBA	+	+	+	−	−	−
IOP before 2nd RBA	----------	-------------	−	--------------	------------	−
OPA before 1st RBA	−	+	+	+	+	+
OPA before 2nd RBA	----------	-------------	−	--------------	------------	−
Lens status	−	−	−	−	−	−
Vitreous status	−	−	−	−	−	−
Axial length [mm]	−	+	+	−	−	−
Lid area [cm^2^]	−	−	−	−	−	−
Eye volume [cm^3^]	−	+	−	−	−	−
Orbital volume [cm^3^]	−	−	−	−	−	−
Orbit/Eye	−	+	+	−	−	−
Anterior chamber depth [mm]	−	−	+	−	−	−
Glaucoma	−	-------------	−	−	−	−
Age [years]	−	−	−	−	−	−
Hearth rate [beats/minute]	−	−	−	−	−	−
Body mass index [kg/m^2^]	+	−	−	−	−	−
Midazolam administration	+	+	−	−	−	−
Midazolam dosage [mg]	+	−	−	−	−	−
Mean arterial pressure before midazolam application [mmHg]	−	−	−	−	+	+
Diabetes mellitus	−	−	−	+	−	−
	Relative change IOP			Relative change OPA		
VAT 0–1	−	+	−	−	−	−
VAT 2–3	−	-------------	------------	−	-------------	-------------

**Table 7:** Overview of multiple linear regression from influencing factors on the change in intraocular pressure (IOP).

Included Parameters	*R* ^2^	Regression Coefficient *B*	*p*	95% *KI*	*n*
Difference IOP before/after 1st RBA [mmHg]					
IOP before 1st RBA [mmHg]	0.3	0.5	<0.001	0.3–0.6	64
Midazolam dosage [mg]	0.4	−3.8	0.02	−6.8–−0.7	63
Difference IOP before 1st/after 2nd RBA [mmHg]					
Axial length [mm]	0.6	2.9	0.001	1.3–4.5	13
Difference IOP before/after 2nd RBA [mmHg]					
IOP before 1st RBA [mmHg]	0.6	1.0	0.001	0.5–1.4	11

**Table 8:** Overview of multiple linear regression from influencing factors on the change in ocular pulse amplitude (OPA).

Included Parameters	*R* ^2^	Regression Coefficient *B*	*p*	95% *KI*	*n*
Difference OPA before/after 1st RBA [mmHg]					
OPA before 1st RBA [mmHg]	0.3	0.6	<0.001	0.4–0.8	69
Diabetes mellitus	0.4	0.7	0.0	0.0–1.4	68
Difference OPA before 1st/after 2nd RBA [mmHg]					
OPA before 1st RBA [mmHg]	0.5	0.8	0.0	0.3–1.4	13
Mean arterial pressure before midazolam [mmHg]	0.6	0.1	0.0	0.0–0.1	12
Difference OPA before/after 2nd RBA [mmHg]					
Mean arterial pressure before midazolam [mmHg]	0.5	0.1	0.0	0.0–0.2	11

## Data Availability

The data presented in this study are available on request from the corresponding author due to data protection.
